# The Effects of Fingolimod (FTY720) on Leukocyte Subset Circulation cannot be Behaviourally Conditioned in Rats

**DOI:** 10.1007/s11481-024-10122-0

**Published:** 2024-05-11

**Authors:** Marie Jakobs, Tina Hörbelt-Grünheidt, Martin Hadamitzky, Julia Bihorac, Yasmin Salem, Stephan Leisengang, Uwe Christians, Björn Schniedewind, Manfred Schedlowski, Laura Lückemann

**Affiliations:** 1 Institute of Medical Psychology and Behavioral Immunobiology, Center for Translational Neuro- & Behavioral Sciences, University Hospital Essen, 45147 Essen, Germany; 2 Department of Clinical Neuroscience, Osher Center for Integrative Medicine, Karolinska Institutet, Stockholm, 171 77 Sweden; 3 iC42 Clinical Research and Development, Department of Anesthesiology, School of Medicine, University of Colorado Anschutz Medical Campus, Aurora, CO USA

**Keywords:** Taste-immune Associative Learning, Fingolimod, Conditioned Taste Avoidance, Immunosuppression

## Abstract

Suppression of immune functions can be elicited by behavioural conditioning using drugs such as cyclosporin A or rapamycin. Nevertheless, little is known about the underlying mechanisms and generalisability of this phenomenon. Against this background, the present study investigated whether the pharmacological properties of fingolimod (FTY720), an immunosuppressive drug widely applied to treat multiple sclerosis, can be conditioned in rats by means of taste-immune associative learning. For this purpose, a conditioned taste avoidance paradigm was used, pairing the presentation of a novel sweet drinking solution (saccharin or sucrose) as conditioned stimulus (CS) with therapeutically effective doses of FTY720 as unconditioned stimulus (US). Subsequent re-exposure to the CS at a later time point revealed that conditioning with FTY720 induced a mild conditioned taste avoidance only when saccharin was employed as CS. However, on an immunological level, neither re-exposure with saccharin nor sucrose altered blood immune cell subsets or splenic cytokine production. Despite the fact that intraperitonally administered FTY720 could be detected in brain regions known to mediate neuro-immune interactions, the present findings show that the physiological action of FTY720 is not inducible by mere taste-immune associative learning. Whether conditioning generalises across all small-molecule drugs with immunosuppressive properties still needs to be investigated with modified paradigms probably using distinct sensory CS. Moreover, these findings emphasize the need to further investigate the underlying mechanisms of conditioned immunomodulation to assess the generalisability and usability of associative learning protocols as supportive therapies in clinical contexts.

**Graphical Abstract:**

**Figure:**
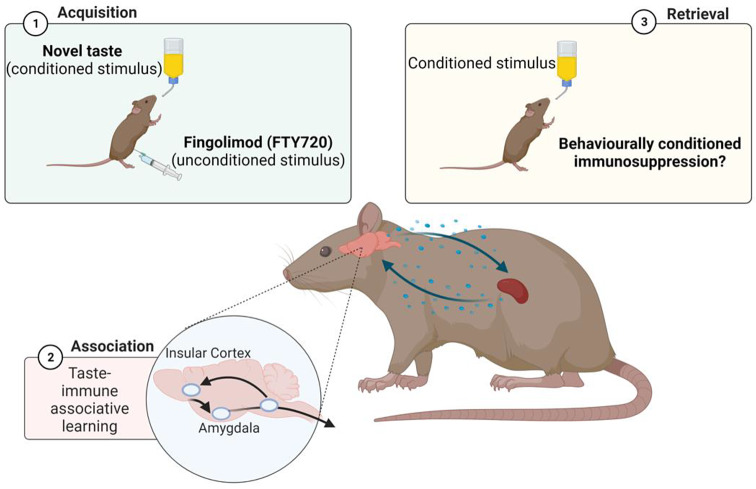
To assess the potential of taste-immune associative learning mediated by specific brain regions (e.g., insular cortex, amygdala), the presentation of a novel taste (saccharin or sucrose) as conditioned stimulus was paired with injections of fingolimod (FTY720) as unconditioned stimulus. However, re-exposure to the conditioned stimulus did not elicit behaviourally conditioned immunosuppressive effects. Created with BioRender.com.

**Supplementary Information:**

The online version contains supplementary material available at 10.1007/s11481-024-10122-0.

## Introduction

The central nervous system (CNS) is able to regulate peripheral immune functions as well as to receive and process signals from the immune system (Hadamitzky et al. 2020; Schiller et al. 2021). A fascinating example for this bidirectional communication between both systems is the behavioural conditioning of immune responses (Hadamitzky et al. 2020). Learned immune responses are, amongst others, inducible via a taste-immune associative learning paradigm in rats, where a novel sweet tasting solution (conditioned stimulus; CS) is paired with the injection of an immunomodulatory drug (unconditioned stimulus; US). Re-exposure to the CS most commonly results in a reduced consumption of the CS (conditioned taste avoidance; CTA). More importantly, CS re-exposure evokes learned alterations of immune functions, similar to those formerly induced by the immunomodulatory drug administered as US (Hadamitzky et al. 2016; Hörbelt et al. 2019; Lückemann et al. 2019). Employing a taste-immune associative learning paradigm, the immunosuppressive properties of substances such as cyclophosphamide (CY), the calcineurin inhibitor cyclosporine A (CsA) as well as the anti-proliferative capacities of the mechanistic target of rapamycin (mTOR) antagonist rapamycin have already been successfully conditioned in rats (Ader and Cohen 1975; von Horsten et al. 1998a; Niemi et al. 2006; Lückemann et al. 2019; Hadamitzky et al. 2020; Leisengang et al. 2022). These learned immune responses are of clinical relevance since they attenuated the progression of autoimmune diseases, prolonged survival of transplanted solid organs and reduced tumor growth in pre-clinical studies (Klosterhalfen and Klosterhalfen 1983; Grochowicz et al. 1991; Lückemann et al. 2020; Hetze et al. 2022). Although not completely understood, conditioned immune responses seem to be mediated via sympathetic innervation of lymphoid organs and beta-adrenoceptor dependent mechanisms (Exton et al. 1999, 2002). Importantly, this phenomenon has been shown to be inducible in healthy humans (Goebel et al. 2002) and could be successfully added to the standard therapy with CsA or tacrolimus in renal transplant patients, thereby amplifying the immunosuppressive effects (Kirchhof et al. 2018). Based on these reports, it has been hypothesised that taste-immune associative learning protocols may be implemented as supportive therapeutic strategies in clinical contexts to maximize the patients´ therapeutic benefit (Albring et al. 2014; Hadamitzky and Schedlowski 2022).

However, whether learned immune responses can be induced by almost all immunomodulating agents or are restricted to a certain class of substances with specific immunopharmacological properties is still unclear (Hadamitzky and Schedlowski 2022). Against this background, the present study investigated whether the immunopharmacological capacity of fingolimod (FTY720), a functional sphingosine-1-phosphate receptor 1 (S1PR1) antagonist, can be behaviourally conditioned by using an established taste-immune associative learning paradigm in rats. FTY720 prevents the egress of lymphocytes from the secondary lymphoid organs into the peripheral blood (Brinkmann et al. 2010) and is widely in clinical use, e.g. for the treatment of multiple sclerosis (Brinkmann 2009; Chun et al. 2021).

## Materials and Methods

### Animals

A total number of 97 male Dark Agouti (DA) rats (202–261 g; Janvier, France) was used in this study. Rats were single housed on an inverse 12 h light/dark cycle (lights off at 7 a.m.) with unlimited access to food and tap water until the experiments started.

### Drug Administration and Sweet Solutions

The dose of FTY720 was chosen based on former studies (Serdar et al. 2016; Herz et al. 2018) and pilot experiments (Online Resource 1, Supplementary Fig.S1) demonstrating that drug administration of 1 mg/kg three times every 72 h elicited potent immunosuppressive effects. FTY720 (Sigma Aldrich) was dissolved in 0.9% NaCl. FTY720 stock solution (1 mg/ml) and control NaCl vials were stored at -20 °C until used. Animals were administered with an injection volume of 1 ml/kg. Saccharin (10 mM; Sigma Aldrich) solution was prepared according to previous studies (Lückemann et al. 2019; Hetze et al. 2022) and was employed as CS. Since data showed that DA rats develop a more pronounced preference for sucrose compared to saccharin (Tordoff et al. 2008), a second experiment used a sucrose solution (100 mM; Sigma Aldrich) as CS.

### Conditioning Paradigm

**Fig. 1:**
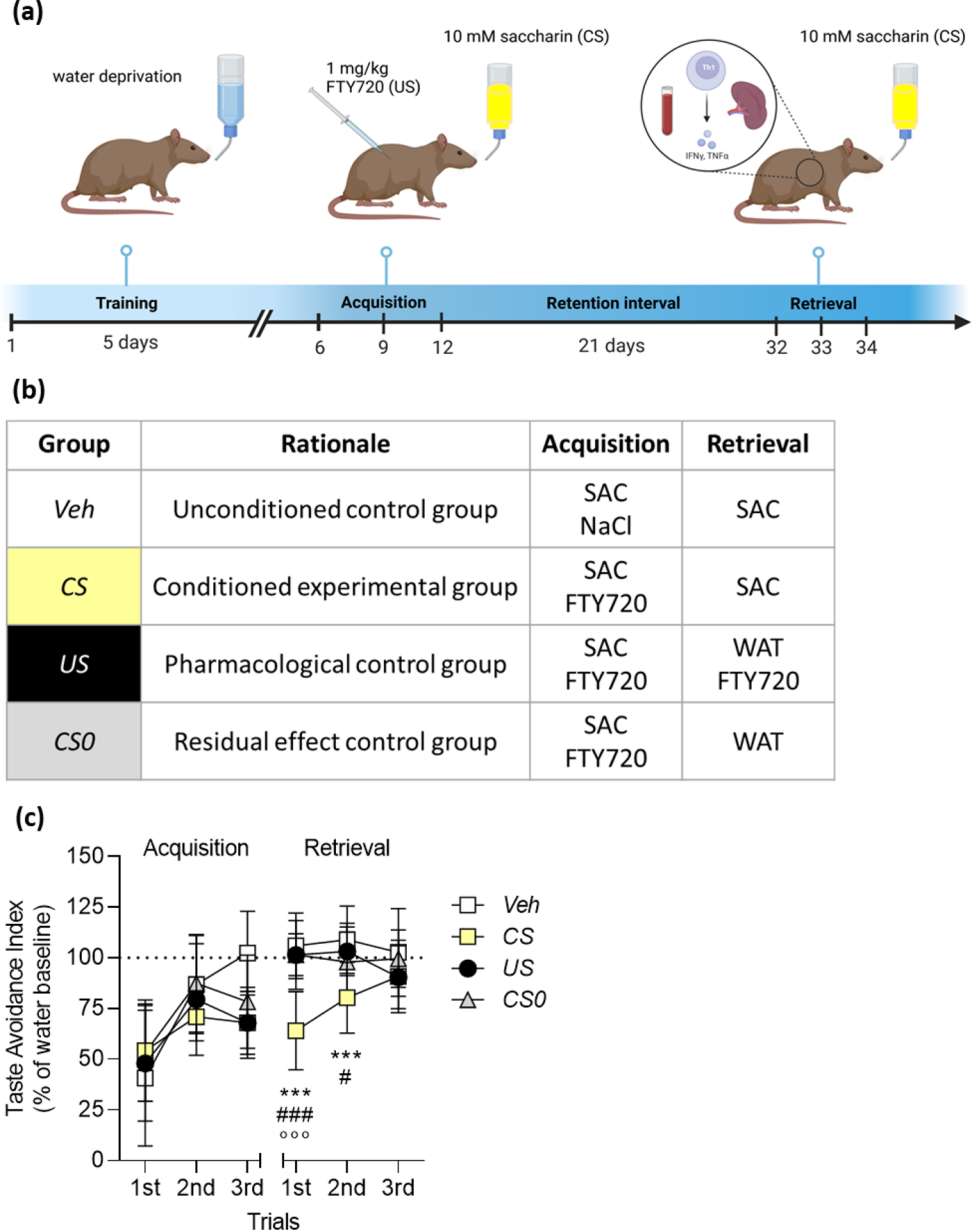
Study design for behavioural conditioning with FTY720 and its impact on the drinking behaviour of rats. (a) Schematic overview of the study design. For acquisition, rats underwent three CS-US (SAC-FTY720) association trials and were re-exposed to the CS during retrieval three times. Created with BioRender.com. (b) Group allocation and treatment design: Animals in the conditioned experimental group (*CS*), residual effect control group (*CS0*), as well as in the pharmacological control group (*US*) were conditioned with SAC and FTY720 during acquisition. During retrieval, animals in the *CS* group and *Veh* group were re-exposed to SAC. The *US* group received water and three FTY720 injections. *CS0* animals were re-exposed to water only (SAC: 10 mM saccharin; FTY720: 1 mg/kg FTY720, i.p.; NaCl: 0.9% NaCl; WAT: tap water; *n* = 8–13/group). (c) During retrieval, re-exposure to saccharin led to a statistically significant CTA in conditioned rats (*CS* group, *n* = 13) compared to the control groups (*Veh*, *n* = 8; *US*, *n* = 11; *CS0*, *n* = 11). Symbols represent a statistically significant difference between groups (ANOVA; Tukey’s test ***p < 0.001, *p < 0.05 (*Veh*) vs. *CS*, ###*p* < 0.001, #*p* < 0.05 (*US*) vs. *CS*, °°°*p* < 0.001 (*CS0*) vs. *CS*). Results are shown as mean percentage changes from water baseline ± SEM

Conditioning was performed based on an established conditioning paradigm (Lückemann et al. 2019; Leisengang et al. 2022) with slight modifications. According to specific pharmacokinetics of FTY720 (i.e., long half-life (Kovarik et al. 2004), the retention interval between acquisition and retrieval was prolonged to 21 days (Fig. 1a). Before the conditioning procedure started, animals were randomly divided into four groups (*CS, US, CS0, Veh*) and set on a water restriction phase for 5 days by allowing them to drink for 15 min at 9 a.m. and 5 p.m. each day. On day six, acquisition started in the morning by presenting the CS, a 10 mM saccharin drinking solution. Subsequently, animals received an intraperitoneal (i.p.) injection of FTY720 (1 mg/kg) as a US. This CS/US pairing was repeated twice more in conditioned (*CS group*), FTY720-treated (*US group*) as well as residual control (*CS0 group*) animals every 72 h. Vehicle controls (*Veh group*) received saccharin together with an i.p. injection of NaCl (1 ml/kg). During the evening sessions and on the days between the acquisition trials all animals received tap water. To measure fluid consumption and to assess CTA, drinking bottles were weighed before and after each drinking session. Following a 21-day retention interval (drug wash-out phase), three retrieval trials were performed separated by 24 h. During retrieval, conditioned rats (*CS group*) received saccharin in the morning and water in the evening. Animals of the *US* group received water together with injections of FTY720 (1 mg/kg) in the morning and water in the evening, serving as pharmacological controls. The *CS0* group only received water during the morning and evening sessions. The *Veh* group was treated like the *CS* group with access to saccharin in the morning and access to water in the evening (Fig. 1b). This conditioning procedure was repeated in a separate series of experiments by using a sucrose solution (100 mM) as CS.

### Splenocyte Isolation

One hour after the last CS re-exposure, deeply anaesthetised animals (isoflurane, 4–5%) were sacrificed by decapitation, spleens were removed and splenocytes were isolated by disrupting the spleen in a Petri dish containing cold HBSS with a 20 ml syringe plunger. The cell suspension was transferred into Falcon tubes and erythrocytes were lysed with diluted BD Pharm Lyse™ (BD Pharmingen). Then, splenocytes were washed in cell culture medium (RPMI, 10% FCS, 1% gentamycin) and filtered through a 70 μm nylon cell strainer. Cell concentrations were determined with an automatic animal cell counter (Vet abc; Medical Solution) and adjusted to a final concentration of 5 × 10^6^ cells/ml.

### Cytokine Analysis

To measure splenic cytokine secretion via Meso Scale Discovery (MSD) multiplex assay, 5 × 10^5^ isolated splenocytes were incubated with 50 ng/ml phorbol myristate acetate (PMA; Sigma Aldrich) and 500 ng/ml ionomycin (Sigma Aldrich) for 24 h at 37 °C and 5% CO_2_. Subsequently, cytokine production was measured in sample supernatants using a V-PLEX Proinflammatory Panel 2 Rat Kit (Meso Scale Discovery) according to manufacturer´s instructions. Additionally, 5 × 10^5^ isolated splenocytes were incubated with 1 µg/ml mouse anti-rat CD3 monoclonal antibody (clone: G4.18; BD Pharmingen) and 1 µg/ml mouse anti-rat CD28 monoclonal antibody (clone: JJ319; BD Pharmingen) for 48 h at 37 °C and 5% CO_2_. To measure IL-17 A concentrations in sample supernatants a commercial enzyme linked immuno-sorbent assay (ELISA Rat IL-17 A; BioLegend) was used according to manufacturer´s instructions. Optical density was assessed on a Fluostar OPTIMA Microplate Reader (BMG Labtech) set to 450 nm. Absolute cytokine concentrations were then calculated using a log–log curve-fit standard curve.

### Blood Leukocyte Analysis

Blood was collected from the tail vein of anaesthetised animals and stored in EDTA monovettes until further processing. Whole blood samples were immunostained for 15 min at RT using fluorescently conjugated antibodies against CD3 (clone: 1F4; BD Biosciences), CD4 (clone: OX-35; BioLegend), CD8 (clone: OX-8; BioLegend), CD11b/c (clone: OX-42; BioLegend), CD45RA (clone: OX-33; BD Biosciences) and CD45 (clone: OX-1; BD Bioscience). Then, blood samples were incubated with eryothrocyte lysis solution (BD Bioscience) for 10 min at RT and washed twice with washing buffer (PBS, 2% FCS, 0.1% NaN_3_). Subsequently, for each sample 20.000 single cells were acquired and analysed by flow cytometry (Celesta; BD Bioscience).

### FTY720 Quantification

To analyse whether i.p. injections of FTY720 can be detected in those brain areas responsible for the association process between the US and the CS, animals (*n* = 12) were injected three times every 72 h with 1 mg/kg FTY720 according to the acquisition phase. Under anaesthesia, 1 h following the last FTY720 injection, tail veine blood was collected in EDTA monovettes and animals were sacrificed by decapitation. Plasma was separated by centrifugation (2,000 x g, 10 min, 4 °C) and stored at -80 °C until further processed. Brains were quickly removed, frozen in isopentan and dry ice, and stored at -80 °C. Using a cryostat microtome (CM1950, Leica), 200 μm thick coronal brain sections were cut at -18 °C and transferred to pre-chilled glass slides. The amygdala (AM) and insular cortex (IC) were then dissected from five serial brain sections using a micropunch technique (Cuello and Carson 1983; Lückemann et al. 2021). Briefly, a pre-chilled stainless steel sample puncher (Ø 2 mm; Fine Science Tools) was used to isolate tissue samples of the left and right IC and AM. Optical tract and hippocampus served as anatomical landmarks to ensure comparable positions of the punched samples across animals (Paxinos and Watson 1998). FTY720 concentration in peripheral EDTA plasma and brain tissue were analysed as described previously with a few modifications (Gottschalk et al. 2011). Briefly, plasma and brain samples were thawed. In addition, brain samples were weighed and homogenised with phosphate buffered saline (pH = 7.4). After protein precipitation with 30% 0.2 M ZnSO_4_ in water/ 70% methanol containing the internal standard fingolimod D_4_, samples were vortexed for 2.5 min and centrifuged (16,000 x g, 10 min). Twenty-five (25) µL supernatant were injected into a 2D high-performance liquid chromatography-tandem mass spectrometry (LC-MS/MS) system for further online extraction and quantification of FTY720. For further details, please see Online Resource 2 (Supplementary Methods).

### Statistical Analysis

Conditioning experiments with saccharin or sucrose were repeated two times with at least *n* = 5 animals per group. Statistical analyses were performed using Sigma Plot (Version 15, Systat Software San Jose, CA, USA) and GraphPad Prism (Version 8). The level of significance was set at *p* < 0.05. The normality of residuals was examined using the Shapiro-Wilk test. Behavioural data (acquisition and retrieval of CTA) were subjected to a two-way analysis of variance (ANOVA) with „group“ (treatment) as a factor and „time“ (days) as a within-subjects factor. Cytokine production and blood immune cell subsets were analysed using one-way ANOVA. Post-hoc individual comparisons between groups were determined by Tukey‘s test, p values were adjusted using the Bonferroni corrections. Merged from two independent experiments, data are shown and evaluated as mean percentage changes from *Veh* controls. Since three animals of the *Veh* group were accidentally presented with water instead of saccharin during the first acquisition, these animals were excluded from behavioural analysis. Animal numbers per treatment group are reported in the figure legends.

## Results

### CTA with Saccharin as CS

During acquisition, no statistically significant differences between groups were detected (time × group interaction (F (6, 78) = 2.2; n.s.; Fig. 1c).

In contrast, during retrieval, ANOVA revealed a significant time × group interaction effect (F (6, 78) = 5.72; *p* < 0.001). Conditioned rats (*CS group*) consumed significantly less saccharin (*p* < 0.001) upon CS re-exposure on retrieval day one compared to all other groups (*Veh, US, CS0*). On retrieval day two, animals of the *CS* group still consumed significantly less compared to *Veh* (*p* < 0.001) and *US* (*p* = 0.007) animals, whereas on retrieval day three no differences between groups were detected (Fig. 1c).

Since DA rats develop a higher preference for sucrose compared to saccharin, a second experiment used sucrose (100 mM) as CS together with FTY720 as US in the aforementioned conditioning paradigm. While our results confirm the overall preference of rats for sucrose, ANOVA revealed no differences in sucrose consumption between groups (Online Resource 1, Supplementary Fig. S2).

### Effect of Behavioural Conditioning with FTY720 on Leukocytes Subset Circulation

Flow cytometry and post-hoc analyses demonstrated that FTY720 treatment led to a statistically significant reduction of peripheral CD3^+^ T cells, CD3^+^CD4^+^ T helper cells, CD3^+^CD8^+^ cytotoxic T cells, CD45RA^+^ B cells (*US group*; *p* < 0.001) compared to the untreated control group (*Veh*). In addition, statistically significant increases in CD11b/c^+^ monocytes and CD11b/c^+^ granulocytes were observed after FTY720 administration (*US group*; *p* < 0.001) compared to controls (*Veh*).

**Fig. 2:**
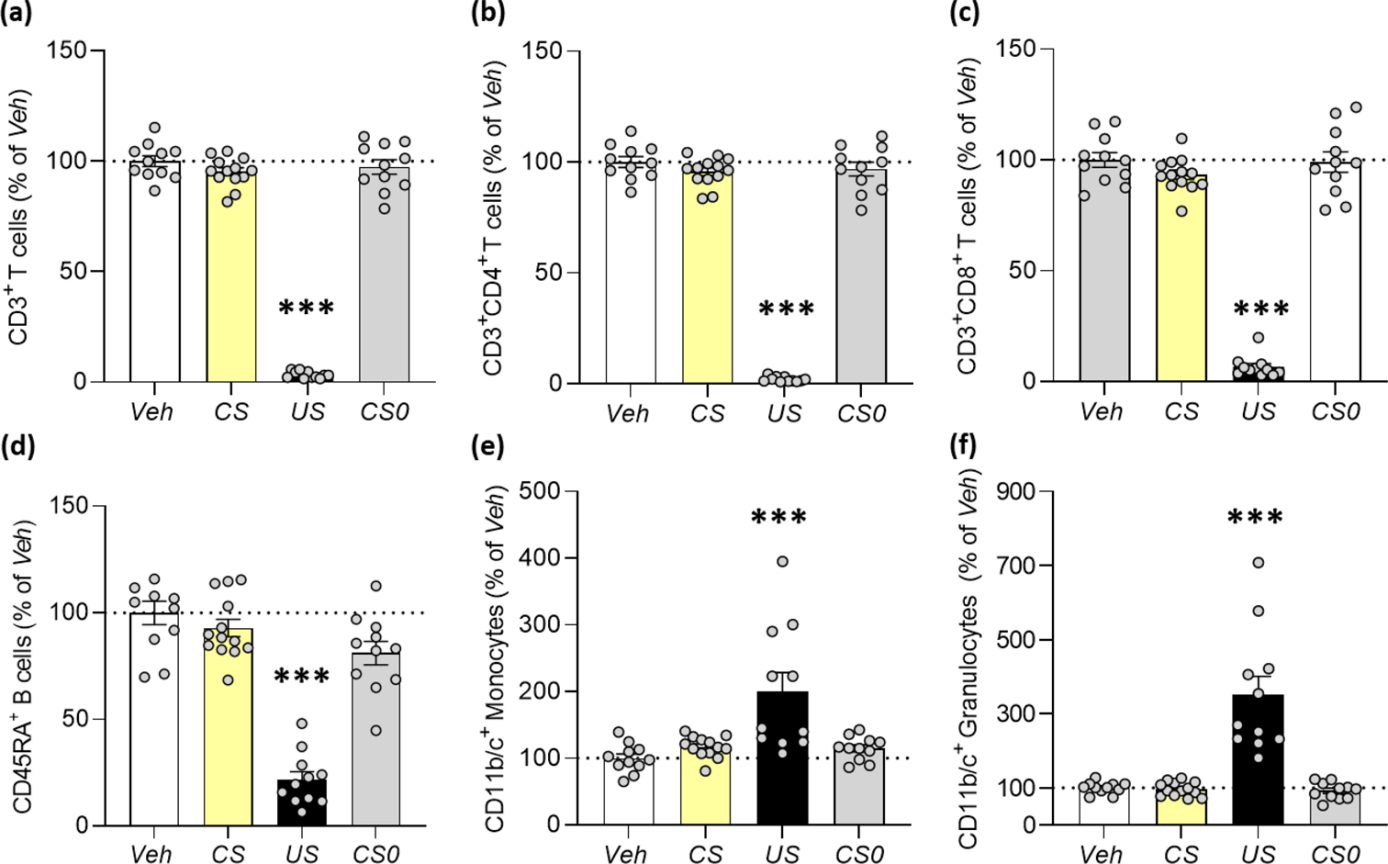
Blood leukocyte count applying a saccharin-FTY720 conditioning paradigm. (a) CD3^+^ T cells, (b) CD3^+^CD4^+^ T cells, (c) CD3^+^CD8^+^ T cells, (d) CD45RA^+^ B cells, (e) CD11b/c^+^ monocytes and (f) CD11b/c^+^ granulocytes were analysed via flow cytometry. While the immunomodulatory effect of FTY720 treatment has been demonstrated (*US*, *n* = 11), no conditioned effects occurred upon saccharin re-exposure (*CS*, *n* = 13) compared to controls (*CS0, Veh*, *n* = 11). Statistical analysis revealed a significant group effect on blood immune cell subset (ANOVA; F (3, 42) = 445.3, *p* < 0.001, Fig. 2a; F (3, 42) = 488.9, *p* < 0.001, Fig. 2b; F (3, 42) = 268.4, *p* < 0.001, Fig. 2c; F (3, 42) = 55.59, *p* < 0.001, Fig. 2d; F (3, 42) = 9.6, *p* < 0.001, Fig. 2e; F (3, 42) = 26.17, *p* < 0.001, Fig. 2f). Asterisks represent a statistically significant difference between groups (ANOVA; Tukey’s test ***p < 0.001 vs. *Veh*; *Veh* = unconditioned control group; *US* = pharmacological control group; *CS* = conditioned experimental group; *CS0* = residual effect control group). Results are shown as mean percentage changes normalised to *Veh* controls ± SEM

In contrast, CS re-exposure during retrieval did not affect the composition of peripheral leukocyte subsets in the *CS group* compared to controls (*CS0, Veh*; n.s.; Fig. 2).

Employing a sucrose-FTY720 paradigm, no behaviourally conditioned alterations of leukocyte subset circulation were observed upon CS re-exposure (*CS group*) compared to controls (*CS0, Veh*) (Online Resource 1, Supplementary Fig. S3).

### Effect of Behavioural Conditioning with FTY720 on Splenic IL-4, IL-5, IL-13 and IL-17 Cytokine Production

Splenic interleukin (IL)-4, IL-5 and IL-17 (*p* < 0.001) as well as IL-13 (*p* < 0.05) cytokine production were substantially reduced in the pharmacological control group (*US*) compared to control animals (*Veh*).

**Fig. 3:**
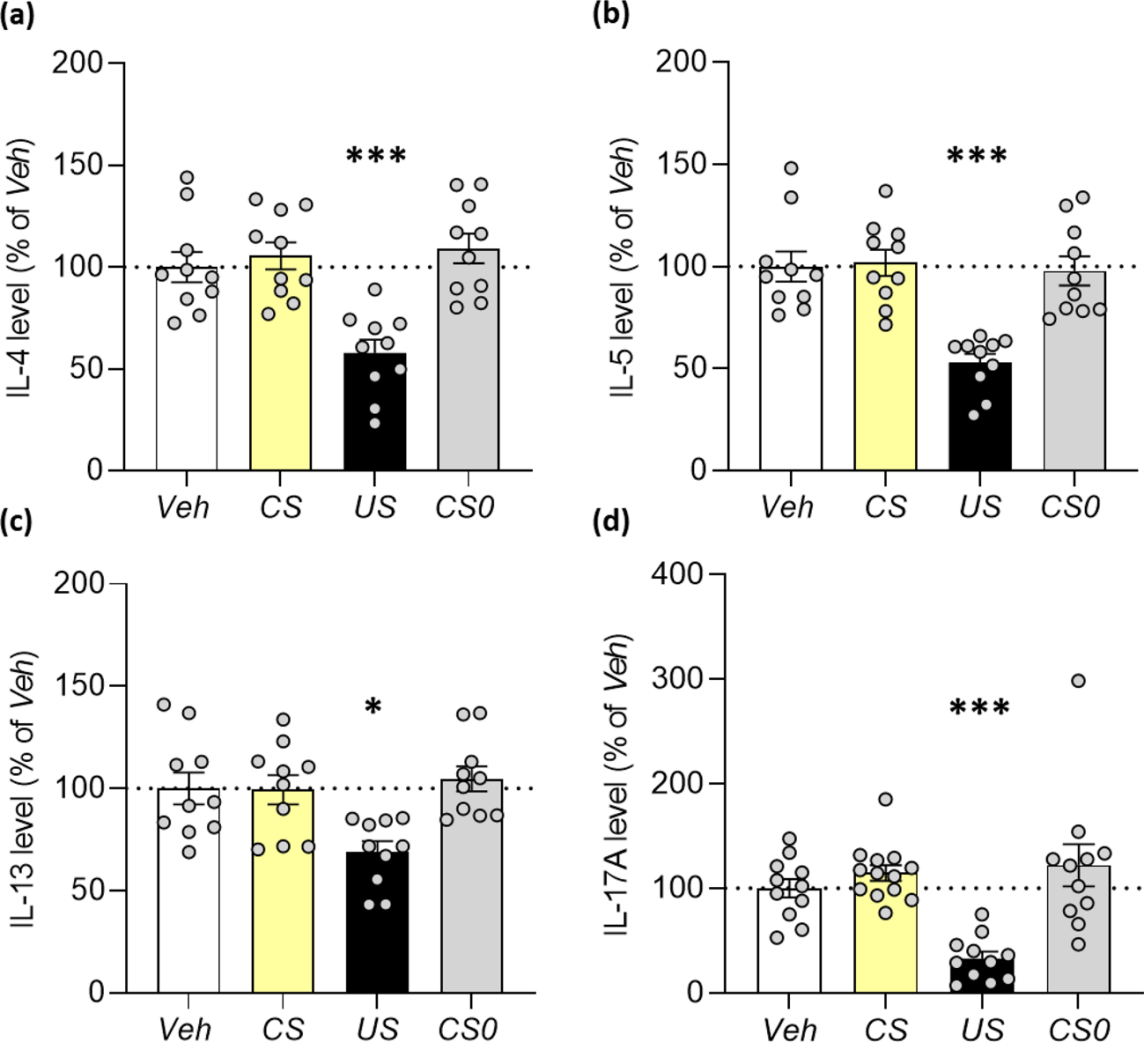
Splenic cytokine secretion applying a saccharin-FTY720 conditioning paradigm. After CS re-exposure, isolated splenocytes were stimulated with (a-c) 50 ng/ml PMA and 500 ng/ml ionomycin for 24 h or with (d) 1 µg/ml CD3 antibody and 1 µg/ml CD28 antibody for 48 h. Cytokine production was measured in the supernatants via (a-c) MSD multiplex assay or via (d) ELISA. FTY720 treatment led to a significant reduction in IL-4, IL-5, IL-13 and IL-17 secretion (*US*, *n* = 10–11). Beyond that, no conditioned immunosupression could be observed in the *CS* group (*n* = 10–13) compared to control groups (*CS0, Veh*, *n* = 10–11). Statistical analysis showed an effect for IL-4, IL-5, IL-13 and IL-17 cytokine production among groups (ANOVA; F (3, 36) = 11.6, *p* < 0.001, Fig. 2a; F (3, 36) = 18.54, *p* < 0.001, Fig. 2b; F (3, 36) = 6.03, *p* = 0.002, Fig. 2c; F (3, 42) = 23.79, *p* < 0.001, Fig. 2d). Asterisks represent a statistically significant difference between groups (ANOVA; Tukey’s test *p < 0.05, ***p < 0.001 vs. *Veh*; *Veh* = unconditioned control group; *US* = pharmacological control group; *CS* = conditioned experimental group; *CS0* = residual effect control group). Results are shown as mean percentage changes normalised to *Veh* controls ± SEM

However, no conditioned reduction of these cytokines in rats of the *CS* group could be observed upon saccharin re-exposure compared to controls (*Veh, CS0*) (Fig. 3).

Using MSD technology, splenic interferon (IFN)-γ, IL-1β, IL-6, keratinocyte chemoattractant (KC)/human growth-regulated oncogene (GRO), IL-10 and tumor necrosis factor (TNF)-α concentrations were measured as well. FTY720 treatment resulted in a statistically significant increase in IL-1β and KC/GRO (*US group*). However, no behaviourally conditioned effects (*CS group*) upon saccharin re-exposure were observed (Online, Resource 1, Supplementary Tab.S1).

Additionally, in the sucrose-FTY720 paradigm no conditioned alterations of splenic cytokine concentrations upon CS re-exposure (*CS group*) were observed compared to controls (*CS0, Veh*) (Online Resource 1, Supplementary Fig. S4).

### FTY720 Quantification

**Fig. 4:**
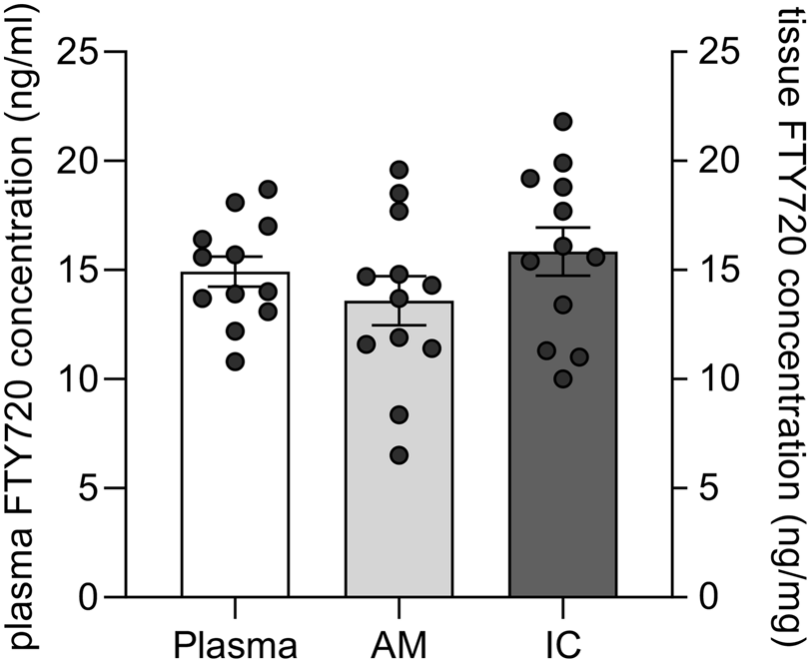
Quantification of FTY720 concentration in plasma, amygdala and insular cortex using high-performance liquid chromatography-tandem mass spectrometry (LC-MS/MS). ﻿Rats (n = 12) were i.p. injected with 1 mg/kg FTY720 three times every 72 h. Blood and brains were collected 1 h after the last injection. After isolating plasma, the amygdala (AM) and the insular cortex (IC), FTY720 concentrations were quantified using LC-MS/MS﻿

Quantification of FTY720 in blood and brain regions known to mediate neuro-immune interactions (AM, IC) using LC-MS/MS verified measurable concentrations of FTY720 in these tissues following i.p. administration (Fig. 4).

## Discussion

Applying an established taste-immune associative learning paradigm in male DA rats, the present study investigated whether and to what extent the immunopharmacological properties of FTY720, a functional S1PR1 antagonist, can be conditioned. On the behavioural level, conditioning with saccharin as CS and FTY720 as US induced a moderate CTA upon CS re-exposure, whereas conditioning with sucrose as CS did not. However, on the immunological level, re-exposure neither to saccharin nor to sucrose during retrieval did affect leukocyte subset distribution or cytokine concentrations in the peripheral blood as seen in FTY720-treated animals. These findings indicate that the immunosuppressive properties of FTY720 could not be behaviourally conditioned, at least with the unique paradigm of taste-immune associative learning.

Previous work has demonstrated that paradigms of taste-immune associative learning in rodents using immunomodulatory substances such as CY, CsA or rapamycin as US can induce learned immune responses mimicking those of the pharmacological drug (Ader and Cohen 1975; von Horsten et al. 1998a; Niemi et al. 2006; Lückemann et al. 2019; Hadamitzky et al. 2020; Leisengang et al. 2022). These behaviourally conditioned immune responses are of clinical relevance since they ameliorated symptomatology of autoimmune diseases, prolonged survival of transplanted organs, and prevented tumor growth in pre-clinical studies (Klosterhalfen and Klosterhalfen 1983; Grochowicz et al. 1991; Hadamitzky et al. 2016; Lückemann et al. 2020; Hetze et al. 2022). More importantly, taste-immune associative learning suppressed lymphocyte proliferation and cytokine production in healthy volunteers (Goebel et al. 2002; Albring et al. 2014) and amplified efficacy of immunosuppressive medication in patients who have had kidney transplants (Kirchhof et al. 2018).

Unfortunately, the present findings show that the physiological action of the immunosuppressive drug FTY720 is not inducible by protocols of taste-immune associative learning. The rather moderate CTA induced by FTY720, which extinguished on retrieval day three, may suggest that the drug might be not salient enough to establish a pronounced CS-US association. In fact, previous data have already shown that the development of a CTA is characteristic for taste-immune associative learning, but not mandatory to retrieve conditioned immune responses (Ader and Cohen 1993; Lückemann et al. 2019). Additionally, numerous studies did report no correlation between the degree of CTA and the degree of conditioned immunosuppression (Bovbjerg et al. 1987; Gorczynski and Kennedy 1987). Moreover, taste-immune associative learning in mice resulted in a conditioned immunosuppression even though animals did not show evidence of avoidance behaviour towards the CS (Niemi et al. 2006). Studies in healthy humans and patients also support this hypothesis, indicative by absent CTA during taste-immune associative learning (Goebel et al. 2002; Kirchhof et al. 2018). Thus, taste re-exposure seems to be crucial for learned immune responses (Niemi et al. 2007), however not for the development or degree of CTA. To rule out the possibility that saccharin was too weak as a stimulus to putatively condition effects of FTY720, a second conditioning setup applied sucrose, since data revealed that DA rats prefer sucrose over saccharin (Tordoff et al. 2008). Even though animals consumed twice as much of the sucrose solution compared to the saccharin solution (Online Resource 1, Supplementary Fig.S2), re-exposure to this more preferred CS did neither induce CTA, nor affected leukocyte subset distribution or cytokine production in the blood (Online Resource 1, Supplementary Fig. S3 and S4).

A recent conditioning study with CsA as US demonstrated that a prolonged retention intervall (up to 30 days) between acquisition and retrieval did not lead to the extinction of the learned behavioural and immunological responses (Hörbelt et al. 2019). Nevetheless, the prolonged retention intervall of 21 days used in the present study to avoid residual effects of the drug during retrieval, might have interfered with the learned taste-immune engram. To exclude the possibility of an early extinction of learned immune responses during the retrieval, leukocyte subset distribution was analysed already after the first CS re-exposure. However, no behaviourally conditioned effects were detected (Online Resource 1, Supplementary Fig. S5).

It has already been demonstrated that immunosuppressive drugs used in taste-immune associated learning paradigms (i.e., CY, rapamycin, CsA) lead to neurobehavioural alterations (von Horsten et al. 1998b; Hadamitzky et al. 2014, 2018). In accordance with this, it has been hypothesised that only neurally mediated substances may qualify as unconditioned responses (Eikelboom and Stewart 1982). The binding of FTY720 at many different receptors (S1PR1, S1PR3, S1PR4, S1PR5) on different cell types might impede characterising relevant pharmacological effects, which qualify as unconditioned response. In addition, it is not clear, whether FTY720 has a similar impact on behaviour and thus on behaviour-associated learning at all. Although i.p. administered FTY720 passes the blood-brain barrier (Cipriani et al. 2017) and accumulates in brain regions mediating taste-immune associative learning (Ramirez-Amaya and Bermudez-Rattoni 1999; Hadamitzky et al. 2020), the impact of FTY720 on behaviour in healthy subjects is still not well characterised (Krivinko et al. 2022).

It is well known that FTY720, CY, CsA as well as rapamycin differ in their ways of inducing immunosuppression. One, perhaps crucial, difference is that FTY720 acts as a selective immunosuppressant by inducing a functional lymphopenia without lymphotoxic action (Ingwersen et al. 2012; Gajofatto et al. 2015). In contrast, CY, CsA and rapamycin are broad immunosuppressants with general destructive/anti-proliferative effects on lymphocytes (Dumont and Su 1996; Matsuda and Koyasu 2000; Ogino and Tadi 2024). The systemic destructive/anti-proliferative effect might lead to the release of cytokines or other molecules resulting in an immediate impact on the vegetative nervous system and other sensing tissue structures, which may be one important factor for successful conditioning.

Taken together, the present data show that the immunomodulatory properties of FTY720 cannot be behaviourally conditioned employing an established and widely used taste-immune associative learning paradigm in rats. These findings once more highlight the complexity of conditioned pharmacological responses in general and show the importance of investigating this phenomenon further with modified paradigms probably using distinct sensory CS, different inter-stimulus intervals, and/or different numbers of acquisition and retrieval trials. Moreover, this study emphasizes the need to further investigate the underlying mechanisms of conditioned immunomodulation to assess the potential generalisability and usability of associative learning protocols as supportive therapies in clinical contexts with the aim to reduce drug doses and thus negative side effects, while maintaining drug efficacy and maximizing the patient´s quality of life (Hadamitzky and Schedlowski 2022).

## Electronic Supplementary Material

Below is the link to the electronic supplementary material.

Supplementary Material 1

Supplementary Material 2

## Data Availability

The datasets generated and analysed during the current study are available from the corresponding author upon reasonable request.
