# Supplementary material for: The Effects of Fingolimod (FTY720) on Leukocyte Subset Circulation cannot be Behaviourally Conditioned in Rats
Source: J Neuroimmune Pharmacol. 2024 May 11;19(1):18. doi: 10.1007/s11481-024-10122-0 (PMC11088542; doi:10.1007/s11481-024-10122-0)

The effects of fingolimod (FTY720) on leukocyte subset circulation cannot be behaviourally conditioned in rats

Journal of Neuroimmune Pharmacology

Marie Jakobs[1*], Tina Hörbelt-Grünheidt[1], Martin Hadamitzky[1], Julia Bihorac[1], Yasmin Salem[1], Stephan Leisengang[1], Uwe Christians[3], Björn Schniedewind[3], Manfred Schedlowski[1,2], Laura Lückemann[1]

[*]Corresponding author:

Marie Jakobs, Institute of Medical Psychology and Behavioral Immunobiology, Center for Translational Neuro- & Behavioral Sciences, University Hospital Essen, 45147 Essen, Germany; E-Mail: Marie.Jakobs@uk-essen.de

Supplementary Methods

LC-MS/MS analysis of FTY720 in rat EDTA plasma and brain tissue punches.

FTY720 was quantified in rat EDTA plasma and brain tissue samples using high-performance liquid chromatography-tandem mass spectrometry (LC-MS/MS) at iC42 Clinical Research and Development (University of Colorado, Aurora, CO, USA). FTY720 (fingolimod) reference material as well as its isotope-labeled internal standard fingolimod-D_4 were from Toronto Research Chemicals (North York, ON, Canada).

Brain tissue punches were weighed and resuspended in 200 µL phosphate buffered saline (PBS). Resuspended brain samples, 200 µL plasma sample aliquots together with corresponding calibrators, quality controls, zero and blank samples were extracted by addition of 800 µL protein precipitation solution (30% 0.2 M $ZnSO_4$ in water/ 70% methanol, v/v) containing the internal standard (10 ng/mL fingolimod-D_4). Samples were vortexed for 2.5 min, centrifuged at 4°C and 16,000 g for 10 min. The supernatants were transferred into 2 mL glass HPLC injection vials. The samples were then further extracted online and analyzed using a 2D-LC-MS/MS system composed of Agilent 1100 HPLC components (Agilent Technologies, Santa Clara, CA, USA) and a Sciex 5000 MS/MS detector (Sciex, Concord, ON, Canada) connected via a turbo flow electrospray source run in the positive ionization mode. The connections of the HPLC components at the 6-port switching valve (Rheodyne, Cotati, CA, USA) are shown below. Twenty-five (25) µL of the extracted samples were injected onto the online extraction column (Zorbax XDB C8, 5 µm particle size, 4.6 · 50 mm, Agilent Technologies) and were washed with a mobile phase of 60% 0.1% formic acid in HPLC grade water (mobile phase A) and 40% methanol containing 0.1% formic acid (mobile phase B). The flow rate was 3 mL/min. After 0.7 min, the switching valve was activated, and the analytes were eluted in the backflush mode from the extraction column onto a 4.6 · 150 mm analytical column filled with C8 material

of 5 µm particle size (Zorbax XDB C8, Agilent Technologies). The analytes were eluted using a gradient that started with 60% mobile phase B, increased to 98% B within 1.7 min, was held at 98% B for 1.4 min and then the analytical column was re-equilibrated to starting conditions (60% B) for 0.5 min. The flow rate was 1.0 mL/min and the analytical column was kept at 60°C. The MS/MS was run in the multiple reaction mode (MRM) and the following ion transitions were monitored: *m/z*= 308.3 $[M+H]^+$ →255.2 (FTY720, quantifier), *m/z*= 308.3 $[M+H]^+$ →105.2 (FTY720, qualifier) and *m/z*= 312.3 $[M+H]^+$ →259.2 (fingolimod-D_4, internal standard). Delustering potentials were set to 51V and collision energies were set to 23V for the FTY720 quantifier transition and the fingolimod D_4 transition and to 35V for the FTY720 qualifier transition.

FTY720 concentrations were quantified using the calibration curves that were constructed by plotting nominal concentration *versus* analyte area to internal standard area ratios (response) using a quadratic fit and 1/x weighting. All calculations were carried out using the Sciex Analyst Software (version 1.7.3). The analytical range for FTY720 was 0.25 (lower limit of quantification) – 100 ng/mL. All results reported here were from runs that met the following acceptance criteria: 75% of the calibrators had to be within ±15% of the nominal value (except at the lower limit of quantification: ±20%) and 2/3 of the quality controls had to be within ±15% of the nominal value. Imprecision of the results was <15%. Significant carry-over and matrix effects were excluded.

Connections and positions of the switching valve.

The left shows the connections during loading onto the online extraction column and online extraction, the right shows the connections after backflush and during LC-MS/MS analysis.

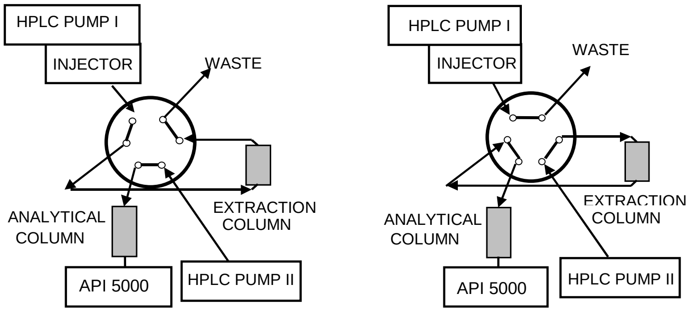

Supplement: Supplementary file 1 — Supplementary Material 1 [file 11481_2024_10122_MOESM1_ESM.pdf]
