# Supplementary material for: The Effects of Fingolimod (FTY720) on Leukocyte Subset Circulation cannot be Behaviourally Conditioned in Rats
Source: J Neuroimmune Pharmacol. 2024 May 11;19(1):18. doi: 10.1007/s11481-024-10122-0 (PMC11088542; doi:10.1007/s11481-024-10122-0)
Supplement: Supplementary file 2 — Supplementary Material 2 [file 11481_2024_10122_MOESM2_ESM.pdf]

# The effects of fingolimod (FTY720) on leukocyte subset circulation cannot be behaviourally conditioned in rats

Journal of Neuroimmune Pharmacology

Marie Jakobs[1*], Tina Hörbelt-Grünheidt[1], Martin Hadamitzky[1], Julia Bihorac[1], Yasmin Salem[1], Stephan Leisengang[1], Uwe Christians[3], Björn Schniedewind[3], Manfred Schedlowski[1,2], Laura Lückemann[1]

**[*]Corresponding author:**

*Marie Jakobs,* Institute of Medical Psychology and Behavioral Immunobiology, Center for Translational Neuro- & Behavioral Sciences, University Hospital Essen, 45147 Essen, Germany; E-Mail: Marie.Jakobs@uk-essen.de

## Supplementary Figures

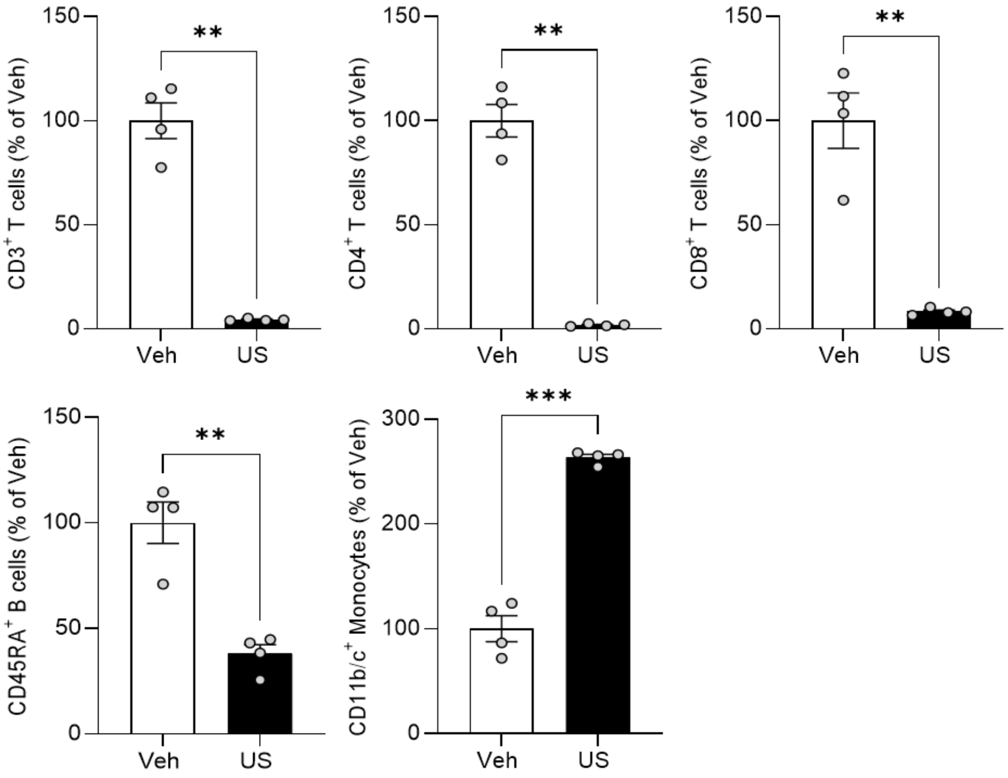


**Supplementary Fig.S1 FTY720 (1 mg/kg) induced a pronounced lymphopenia in rats**

Pilot data that demonstrated that 1 mg/kg FTY720 led to a pronounced lymphopenia. Rats were i.p. injected with 1 mg/kg FTY720 (US, n = 4) or 0.9% NaCl (Veh, n = 4) three times every 72 h. Blood was drawn and analysed via flow cytometry 24 h after the last injection. FTY720 treatment resulted in a reduction of (a) $CD3^+$ T cells, (b) $CD4^+$ T cells, (c) $CD8^+$ T cells, (d) $CD45RA^+$ B cells and in an increase of (e) CD11b/$c^+$ monocytes. Asterisks represent a statistically significant difference between groups (unpaired t test with Welch's correction, **$p < 0.01$, ***$p < 0.001$ vs. *Veh*; *Veh* = control group; *US* = pharmacological group). Results are shown as mean percentage changes normalised to *Veh* controls ± SEM.

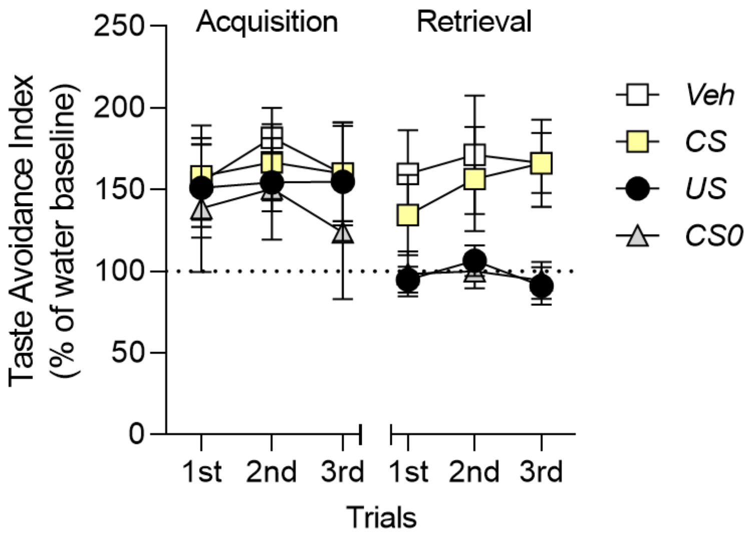


**Supplementary Fig.S2 Rats conditioned with a sucrose-FTY720 conditioning paradigm did not develop a CTA**

Animals in the conditioned experimental group (*CS, n = 10*), residual effect control group (*CS0, n = 10*), as well as in the pharmacological control group (*US, n = 10*) were conditioned with 1 mg/kg FTY720 and 100 mM sucrose during acquisition. During retrieval, re-exposure to sucrose did not induce a CTA in conditioned rats (*CS* group) compared to the *Veh* control group (n = 10). Results are shown as mean percentage changes from water baseline ± SEM.

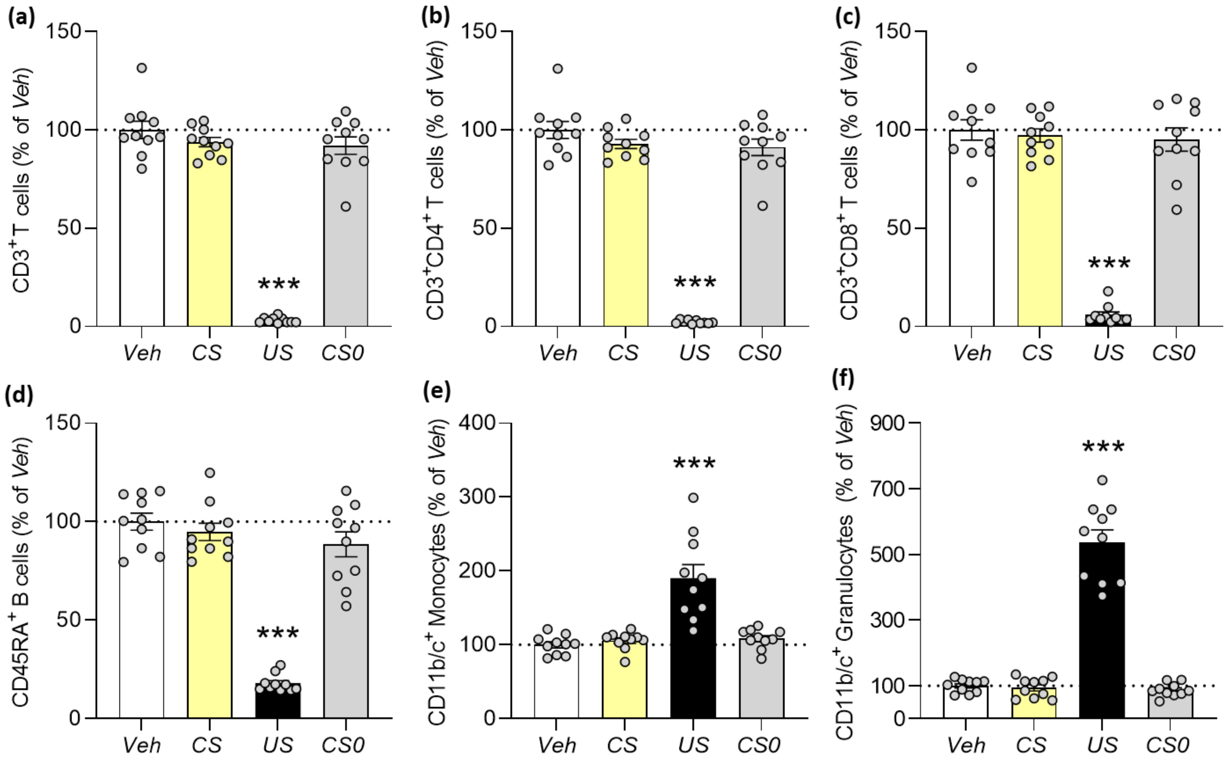


**Supplementary Fig.S3 Blood leukocyte count applying a sucrose-FTY720 conditioning paradigm**

(a) CD3$^+$ T cells, (b) CD3$^+$CD4$^+$ T cells, (c) CD3$^+$CD8$^+$ T cells, (d) CD45RA$^+$ B cells, (e) CD11b/c$^+$ monocytes and (f) CD11b/c$^+$ granulocytes were analysed via flow cytometry. While the immunomodulatory effect of FTY720 treatment has been demonstrated (*US,* n = 10), no conditioned effects occurred upon sucrose re-exposure (*CS,* n = 10) compared to the Veh control (n = 10). Asterisks represent a statistically significant difference between groups (ANOVA; Tukey's test ***p < 0.001 vs. *Veh*; *Veh* = unconditioned control group; *US* = pharmacological control group; *CS* = conditioned experimental group; *CS0* = residual effect control group). Results are shown as mean percentage changes normalised to *Veh* controls ± SEM.

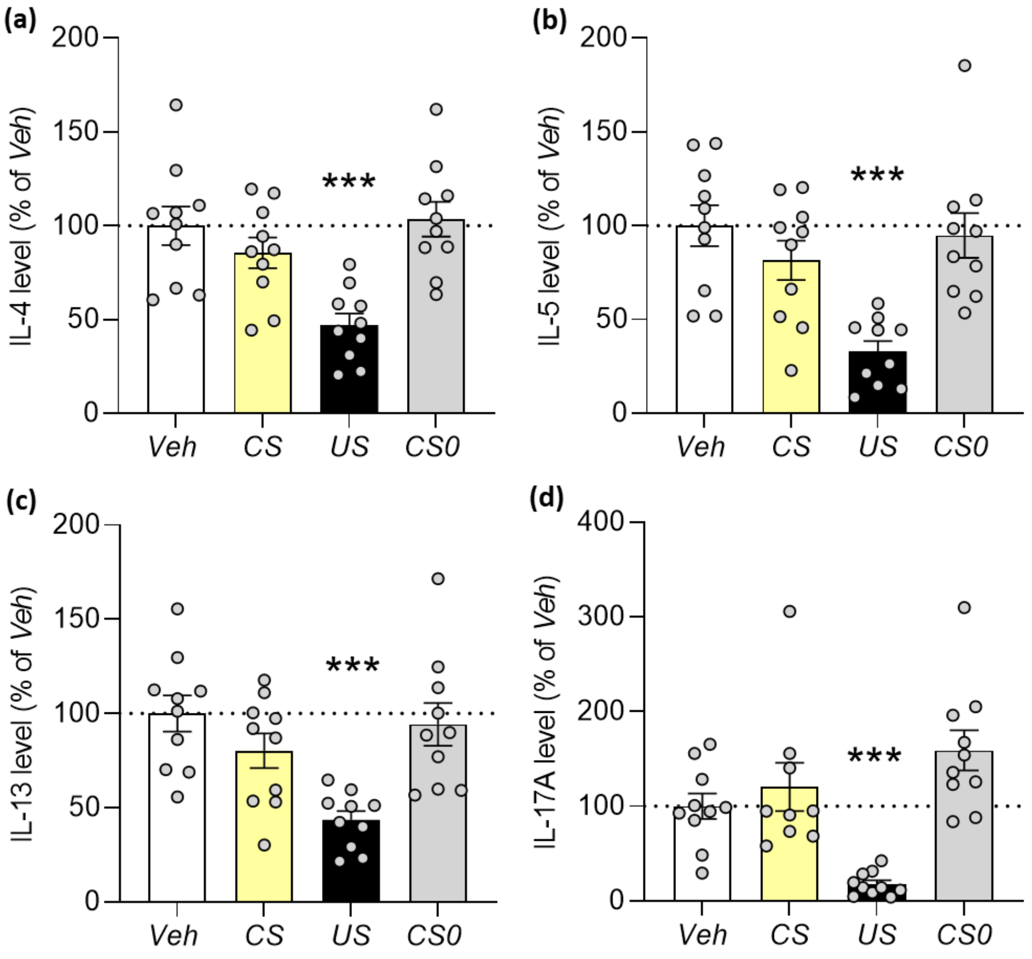


**Supplementary Fig.S4 Splenic cytokine secretion applying a sucrose-FTY720 conditioning paradigm**

After CS re-exposure, isolated splenocytes were stimulated with (a-c) 50 ng/ml PMA and 500 ng/ml ionomycin for 24 h or with (d) 1 µg/ml CD3 antibody and 1 µg/ml CD28 antibody for 48 h. Cytokine production was measured in the supernatants via (a-c) MSD multiplex assay or via (d) ELISA. FTY720 treatment led to a significant reduction in IL-4, IL-5, IL-13 and IL-17 secretion (*US,* n = 10). Beyond that, no conditioned immunosuppression could be observed in the *CS* group (n = 10) compared to the Veh control group (n =10). Asterisks represent a statistically significant difference between groups (ANOVA; Tukey's test ***p < 0.001 vs. *Veh*; *Veh* = unconditioned control group; *US* = pharmacological control group; *CS* = conditioned experimental group; *CS0* = residual effect control group). Results are shown as mean percentage changes normalised to *Veh* controls ± SEM.

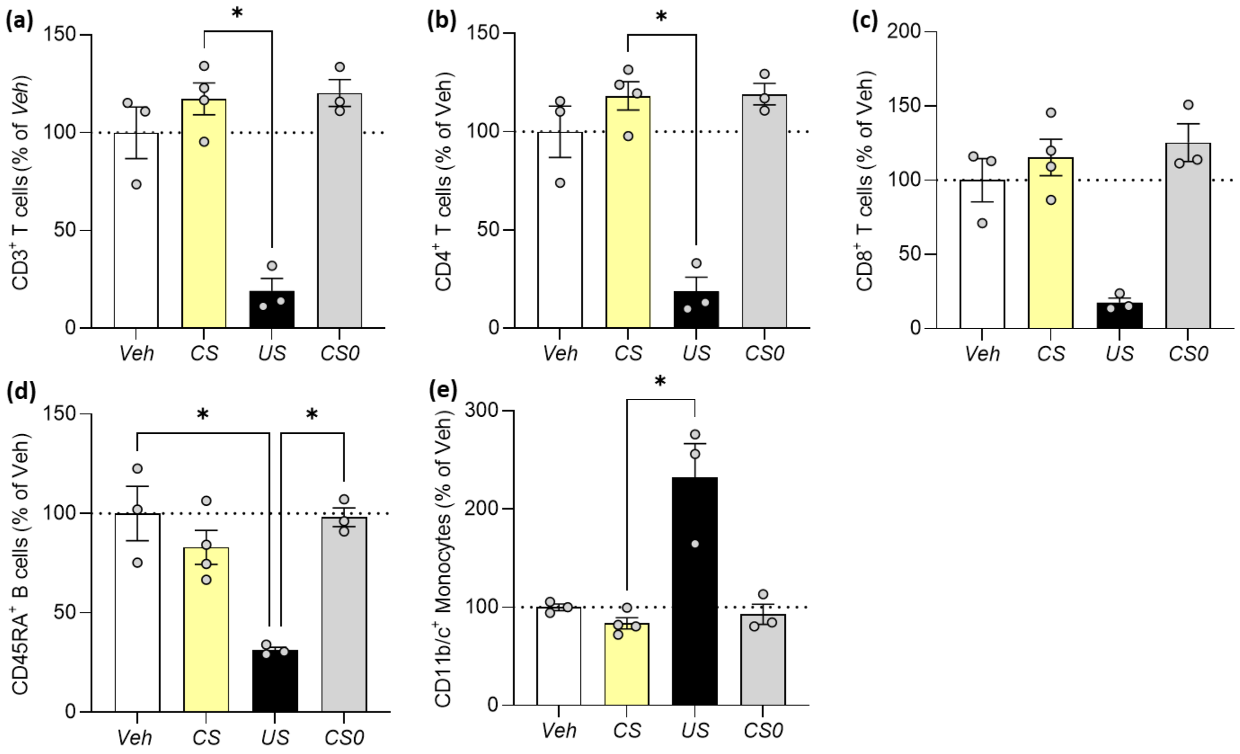


**Supplementary Fig.S5 Single CS re-exposure during retrieval does not induce a behaviourally conditioned lymphopenia in rats**

Pilot data demonstrated that a single CS re-exposure during retrieval does not result in behaviorally conditioned alterations of immune cell subsets. During acquisition, rats were conditioned with 1 mg/kg FTY720 as US and 10 mM saccharin as CS (*CS, US, CS0*, n = 3-4) or i.p. injected with 0.9% NaCl (*Veh*, n = 3) three times every 72 h. Blood was drawn and analysed via flow cytometry 1 h after the first CS re-exposure during retrieval. FTY720 treatment resulted in a reduction of (a) $CD3^+$ T cells, (b) $CD4^+$ T cells, (c) $CD8^+$ T cells, (d) $CD45RA^+$ B cells and in an increase of (e) $CD11b/c^+$ monocytes. However, CS re-exposure did not induce a behaviourally conditioned lymphopenia. Asterisks represent a statistically significant difference between groups (Kruskal-Wallis test, Dunn´s multiple comparisons test, *$p < 0.05$ vs. *US*; *Veh* = control group; *CS* = conditioned group; *US* = pharmacological control group; *CS0* = residual effect control group). Results are shown as mean percentage changes normalised to *Veh* controls ± SEM.

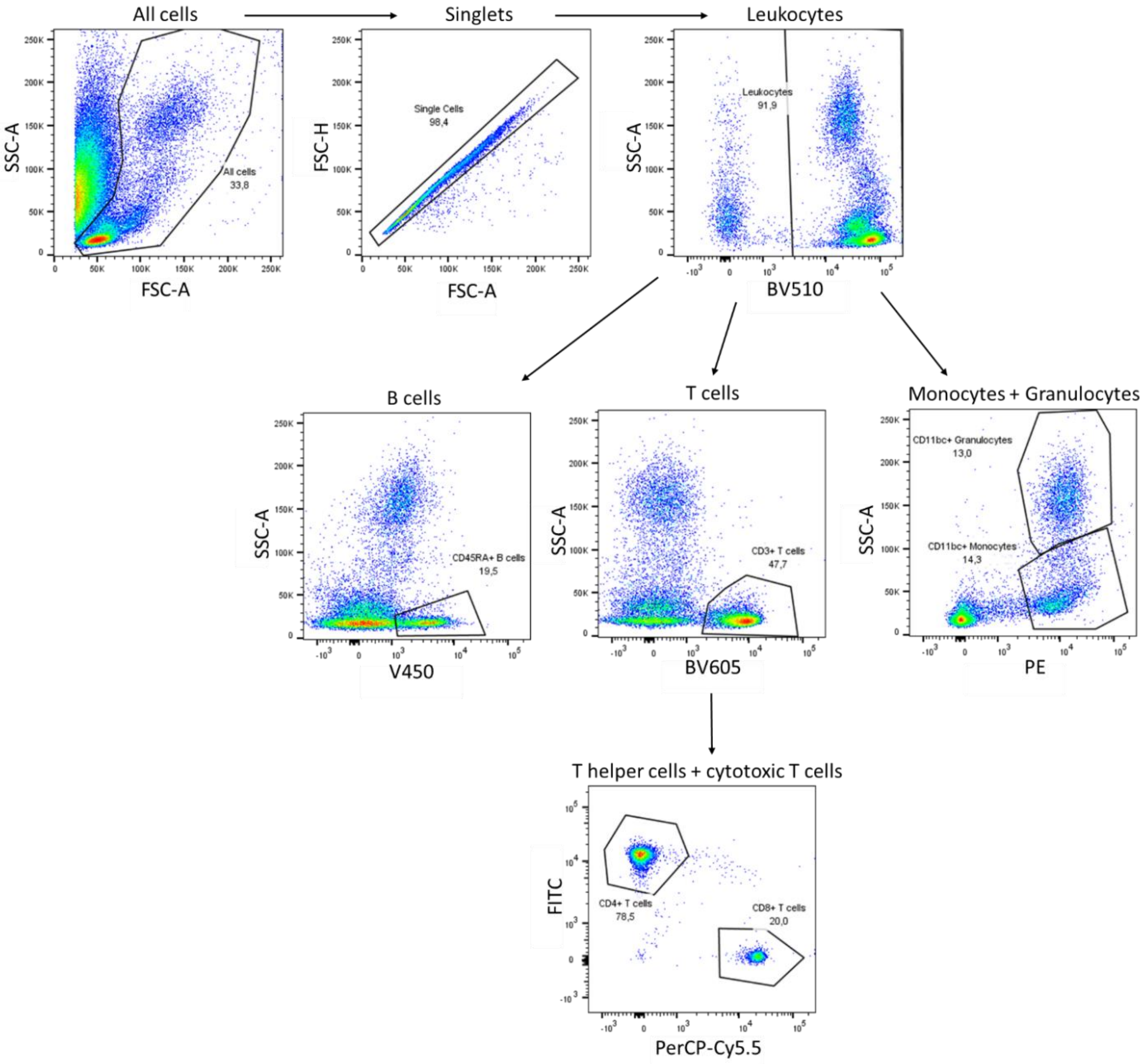


**Supplementary Fig.S6 Flow cytometry gating strategy**

Immunophenotype characterisation of murine peripheral blood cells. Logic and manual gating strategy for immune cell subset characterisation using six monoclonal antibody-conjugated CD markers simultaneously, including CD45-BV510, CD45RA-V450, CD3-BV605, CD11b/c-PE, CD4-FITC and CD8-PerCP-CY5.5.

| cytokine (% of *Veh*) | IFN-y | IL-1ß | IL-6 | KC/ GRO | IL-10 | TNF-a |
|---|---|---|---|---|---|---|
| *CS* | 111.1% (± 7.2%) | 98.2% (± 3.2%) | 122.7% (± 6.6%) | 109.4% (± 4.2%) | 117.1% (± 9.9%) | 87.0% (± 4.8%) |
| *US* | 77.7% (± 8.9%) | 156.5% (± 7.8%) *** | 125.0% (± 6.7%) | 227.9% (± 22.1%) *** | 90.6% (± 9.7%) | 74.7% (± 3.2%) |
| *CS0* | 109.4% (± 10.8%) | 99.1% (± 4.5%) | 114.3% (± 9.9%) | 115.0% (± 11.4%) | 114.2% (± 18.3%) | 92.4% (± 4.1%) |

**Supplementary Tab.S1 Splenic cytokine levels (% of Veh) after applying the saccharin-FTY720 conditioning paradigm**

Using Meso Scale Discovery (MSD) technology, splenic IFN-γ, IL-1β, IL-6, KC/GRO, IL-10 and TNF-α concentration were measured. FTY720 treatment resulted in an increase in IL-1β and KC/GRO (*US group,* n = 10-11) compared to *Veh* controls (n = 10-11). However, no conditioned effects (*CS group,* n = 10-13) upon saccharin re-exposure were observed. Asterisks represent a statistically significant difference between groups (ANOVA; Tukey's test ***p < 0.001 vs. *Veh*; *Veh* = unconditioned control group; *US* = pharmacological control group; *CS* = conditioned experimental group; *CS0* = residual effect control group (n =10-11)). Splenic cytokine levels are depicted as percent of *Veh* ± SEM. *Veh* was set to 100%.
